# Evolution of Immune Evasion and Host Range Expansion by the SARS-CoV-2 B.1.1.529 (Omicron) Variant

**DOI:** 10.1128/mbio.00416-23

**Published:** 2023-04-03

**Authors:** Wenlin Ren, Yu Zhang, Juhong Rao, Ziyi Wang, Jun Lan, Kunpeng Liu, Xuekai Zhang, Xue Hu, Chen Yang, Guocai Zhong, Rong Zhang, Xinquan Wang, Chao Shan, Qiang Ding

**Affiliations:** a Center for Infectious Disease Research, School of Medicine, Tsinghua University, Beijing, China; b State Key Laboratory of Virology, Center for Biosafety Mega-Science, Wuhan Institute of Virology, Chinese Academy of Sciences, Wuhan, Hubei, China; c University of Chinese Academy of Sciences, Beijing, China; d School of Life Sciences, Tsinghua University, Beijing, China; e Shenzhen Bay Laboratory, Shenzhen, China; f School of Chemical Biology and Biotechnology, Peking University Shenzhen Graduate School, Shenzhen, China; g Key Laboratory of Medical Molecular Virology (MOE/NHC/CAMS), School of Basic Medical Sciences, Shanghai Medical College, Biosafety Level 3 Laboratory, Fudan University, Shanghai, China; Virginia Polytechnic Institute and State University

**Keywords:** COVID-19, SARS-CoV-2, Omicron variant, B.1.1.529, host range, ACE2 decoy receptor

## Abstract

Recently, severe acute respiratory syndrome coronavirus 2 (SARS-CoV-2) variant B.1.1.529 (Omicron) has rapidly become the dominant strain, with an unprecedented number of mutations within its spike gene. However, it remains unknown whether these variants have alterations in their entry efficiency, host tropism, and sensitivity to neutralizing antibodies and entry inhibitors. In this study, we found that Omicron spike has evolved to escape neutralization by three-dose inactivated-vaccine-elicited immunity but remains sensitive to an angiotensin‐converting enzyme 2 (ACE2) decoy receptor. Moreover, Omicron spike could use human ACE2 with a slightly increased efficiency while gaining a significantly increased binding affinity for a mouse ACE2 ortholog, which exhibits limited binding with wild-type (WT) spike. Furthermore, Omicron could infect wild-type C57BL/6 mice and cause histopathological changes in the lungs. Collectively, our results reveal that evasion of neutralization by vaccine-elicited antibodies and enhanced human and mouse ACE2 receptor engagement may contribute to the expanded host range and rapid spread of the Omicron variant.

## INTRODUCTION

Since its emergence in late 2019, the severe acute respiratory syndrome coronavirus 2 (SARS-CoV-2) strain that caused the coronavirus disease 2019 (COVID-19) pandemic has been evolving into several new viral variants of concern (VOCs) (1–4). SARS‐CoV‐2 entry into cells is initiated by interactions between the spike glycoprotein (S) and its receptor angiotensin‐converting enzyme 2 (ACE2) in a species-dependent manner (2, 5, 6). Thus, mutations in spike may alter SARS-CoV-2’s transmission, host tropism, pathogenicity, and sensitivity to vaccine-elicited antibodies (7–9). For example, the D614G mutation, identified at an earlier stage of the pandemic, promotes spike binding to ACE2, leading to enhanced virus transmission (10, 11). Subsequently, the N501Y mutation found in the B.1.1.7, B.1.351, and B.1.1.28.1 spike proteins has increased the binding affinity between the receptor-binding domain (RBD) and ACE2, increasing viral fitness and infectivity (1, 12, 13). In addition, K417N and E484K found in the B.1.351 variant contribute to the evasion of neutralization by multiple monoclonal antibodies (14–16). Furthermore, it has been reported that mouse, New World monkey, or koala ACE2 could not bind the SARS-CoV-2 spike protein, thus hindering viral entry into these species (17). Mutations in the S protein could alter its affinity for animal ACE2, and new variants such as B.1.351 (Beta) and B.1.1.529 (Omicron) can infect wild-type (WT) mice (18–20). Thus, as the COVID-19 pandemic continues, it is critical to closely monitor the emergence of new variants as well as their impacts on viral transmission, pathogenesis, and vaccine and therapeutic efficacies.

Recently, the Omicron variant has rapidly become the dominant circulating VOC (https://www.who.int). This variant is highly transmissible and evades a panel of neutralizing antibodies due to a high number of substitutions in the spike glycoprotein (21–23). Omicron carries A67V, Δ69–70, T95I, G142D, Δ143–145, Δ211, L212I, and ins214EPE in the N-terminal domain (NTD) of spike; G339D, S371L, S373P, S375F, K417N, N440K, G446S, S477N, T478K, E484A, Q493K, G496S, Q498R, N501Y, and Y505H in the RBD; P681H in proximity to the furin cleavage site; as well as other mutations (T547K, D614G, H655Y, N679K, N764K, D796Y, N856K, Q954H, N969K, and L981F). Indeed, some of the mutations in Omicron have been present in other variants, for example, Δ69–70 and P681H in the Alpha variant, K417N in the Beta variant, T478K in the Delta strain, and N501Y in the Alpha, Beta, and Gamma strains.

There are numerous medical and scientific questions concerning the Omicron variant that urgently need to be addressed. Here, we evaluated the sensitivity of Omicron to convalescent-phase sera and a soluble ACE2-Ig decoy receptor, and characterized the spike protein of Omicron for its ability to utilize different ACE2 orthologs for cell entry.

## RESULTS

### The Omicron variant exhibited resistance to neutralization by vaccine-elicited sera but remained sensitive to an ACE2 decoy receptor.

**FIG 1:**
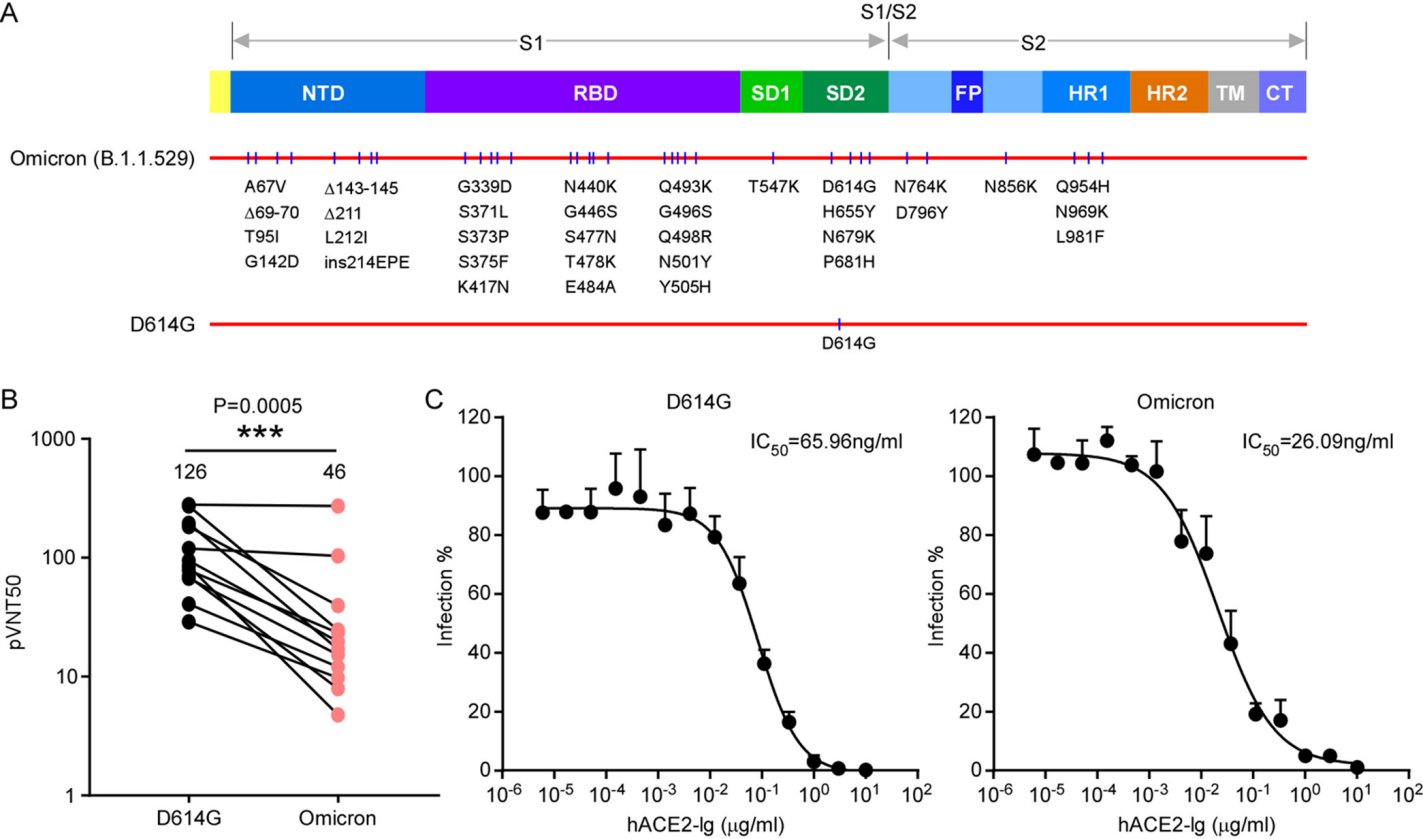
Sensitivity of pseudoviruses bearing D614G or Omicron spike proteins to neutralization by sera from vaccine recipients and an ACE2 decoy receptor. (A) Schematic overview of SARS-CoV-2 spike proteins of Omicron (B.1.1.529), colored by domain. Mutations on the schematic refer to the SARS-CoV-2 Wuhan-Hu-1 isolate (WT) (GenBank accession no. MN908947). NTD, N-terminal domain; RBD, receptor-binding domain; SD1, subdomain 1; SD2, subdomain 2; FP, fusion peptide; HR1, heptad repeat 1; HR2, heptad repeat 2; TM, transmembrane region; CT, cytoplasmic tail. (B) Neutralization by sera from vaccine recipients of D614G and Omicron SARS-CoV-2 pseudovirus infection of HeLa-ACE2 cells. All sera were 3-fold serially diluted. The serum dilution factor leading to a 50% reduction in spike protein-driven cell entry (pVNT_50_) against each pseudovirion was plotted, and identical serum samples are connected with lines. The statistical significance of differences between D614G and variant spike proteins was analyzed by a two-sided Friedman test with Dunn’s multiple-comparison test. *, *P* < 0.05; **, *P* < 0.01; ***, *P* < 0.001. The pVNT_50_ of each sample was tested in two repeats. (C) Recombinant ACE2-Ig was diluted to the indicated concentrations. Viral entry was determined as described above for panel A. The dilution factor leading to a 50% reduction of pseudotyped-virion entry was calculated as the IC_50_ using GraphPad Prism software. The data shown are representative of data from three independent experiments with similar results, and data points represent the means ± standard deviations (SD) in triplicate.

Given that the Omicron variant harbors a large number of mutations in the spike (S) protein (Fig. 1A), which is the target of neutralizing antibody responses against this virus, we sought to determine the sensitivity of this variant to serum samples obtained from persons who had been vaccinated with inactivated SARS-CoV-2 vaccines. We obtained serum samples from healthy persons who had received three doses of the inactivated vaccine Sinovac-CoronaVac or BBIBP-CorV (see Table S1 in the supplemental material). For all serum samples, we determined the titers of neutralizing antibodies against virions pseudotyped with spike proteins from the D614G or Omicron variant. Specifically, we preincubated serially diluted sera from 12 persons with virions pseudotyped with spike proteins as described above and subsequently tested them on HeLa-human ACE2 cells. The cell entry of pseudotyped virions in the presence of sera with various concentrations was assessed 48 h later by measurements of luciferase activities (Fig. 1B and Table S1). Of the serum samples from the 12 persons, 2 exhibited comparable neutralization abilities against both D614G and Omicron infections, and the other 10 exhibited reduced neutralization abilities against Omicron. Collectively, serum samples from vaccinated persons neutralized the Omicron variant with a 3.7-fold reduction compared with D614G (Fig. 1B).

10.1128/mbio.00416-23.6 TABLE S1 Information for serum samples from healthy persons who had received three doses of the inactivated vaccine Sinovac-CoronaVac or BBIBP-CorV. Download Table S1, XLSX file, 0.01 MB. Copyright © 2023 Ren et al. 2023 Ren et al. https://creativecommons.org/licenses/by/4.0/ This content is distributed under the terms of the Creative Commons Attribution 4.0 International license.

Our studies and those of other groups have shown that a soluble ACE2 decoy exhibited a potent antiviral effect against SARS-CoV-2 infection *in vitro* and *in vivo* (24, 25). As Omicron’s spike protein still effectively utilizes human ACE2 for cell entry, it is conceivable that it would be sensitive to inhibition by an ACE2 decoy receptor. To test this, we inoculated HeLa-human ACE2 cells with pseudovirions bearing the D614G or Omicron spike protein in the presence of ACE2-Ig at various concentrations. After 48 h, the cells were collected, and viral entry was assessed. ACE2-Ig could potently inhibit D614G- and Omicron-pseudotyped virion infections with 50% inhibitory concentrations (IC_50_s) of 65.96 ng/mL and 26.09 ng/mL, respectively, indicating that the Omicron is still or even more sensitive to inhibition by ACE2-Ig (Fig. 1C). In summary, Omicron exhibited a reduced sensitivity to neutralization by vaccine-elicited sera but remained sensitive to ACE2 decoy receptor antiviral countermeasures.

### The spike protein of Omicron gained an increased binding affinity for human and mouse ACE2 orthologs.

**FIG 2:**
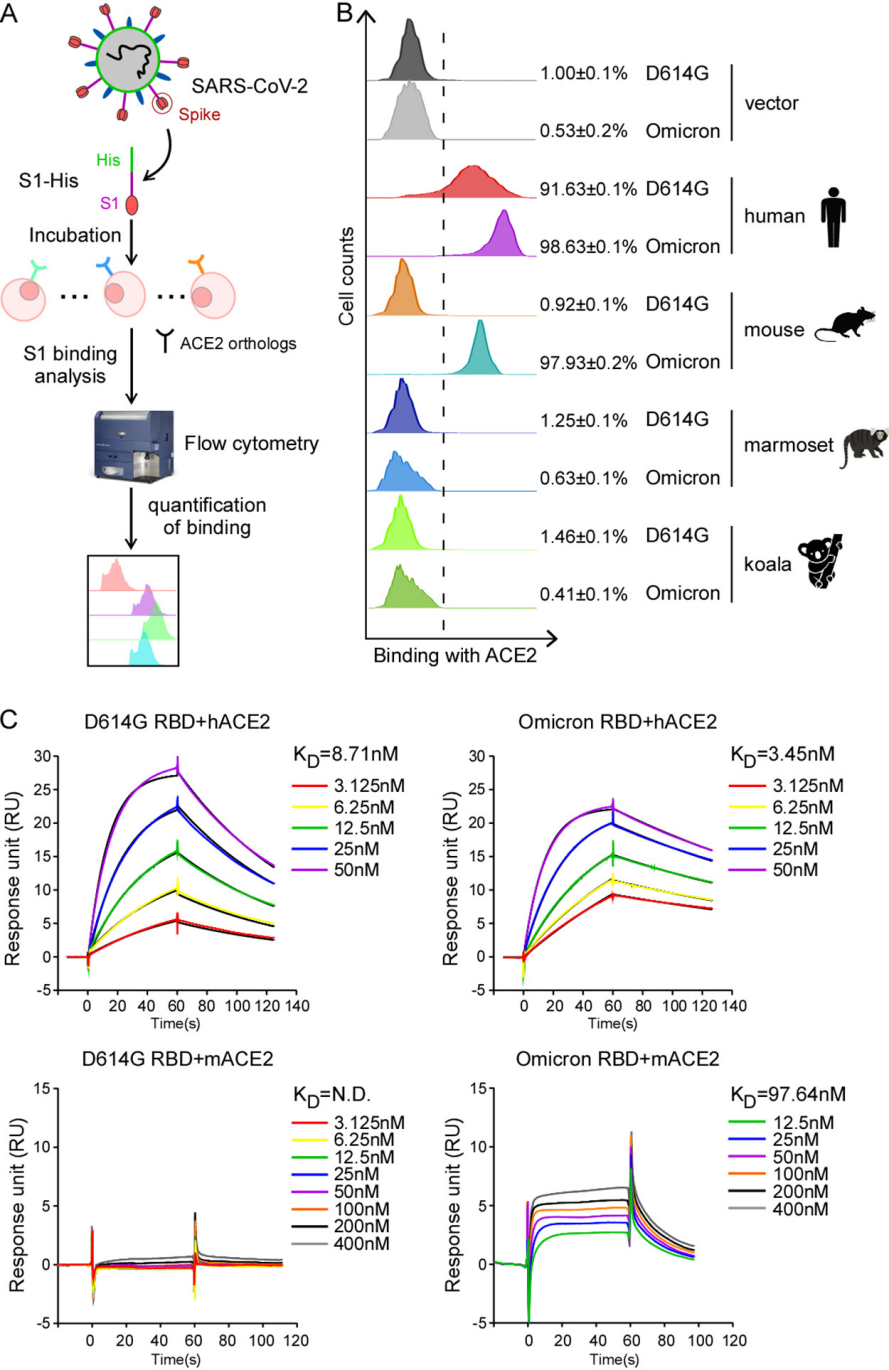
Characterization of the binding of D614G or Omicron spike proteins to ACE2 orthologs. (A) Schematic for testing the efficiency of ACE2 orthologs binding D614G or Omicron S1 proteins. (B) HeLa cells were transduced with human or other ACE2 orthologs as indicated. The transduced cells were incubated with D614G or Omicron S1 in which the C terminus was fused with a His tag and then stained with anti-His-PE for flow cytometry analysis. Values are expressed as the percentages of cells positive for S1-Fc among the ACE2-expressing cells (zsGreen1-positive cells) and shown as the means ± SD from 3 biological replicates. These experiments were independently performed three times, with similar results. (C) The kinetics of binding of human or mouse ACE2 proteins to the recombinant D614G or Omicron RBD were determined using the Biacore system. ACE2 proteins were captured on a chip, and serial dilutions of the RBD were then injected over the chip surface. Experiments were performed three times, with similar results, and one set of representative data is displayed.

The new SARS-CoV-2 variant Omicron emerged and rapidly spread across the world, which could be caused by the increased ability to enter cells since the variants harbor a large number of mutations in the spike gene (Fig. 1A). To examine the biological consequences of these mutations on binding with the ACE2 receptor, we employed a cell-based assay that uses flow cytometry to assess the binding of the RBD of the spike protein to human and animal ACE2s (Fig. 2A). We derived HeLa cells stably expressing human or animal ACE2 orthologs by transduction with a bicistronic lentiviral vector (pLVX-IRES-zsGreen1) that expresses the fluorescent protein zsGreen1 via an internal ribosome entry site (IRES) element, which is used to monitor the transduction efficiency. Next, D614G- or Omicron-derived recombinant S1-His (a fusion protein consisting of the RBD and a polyhistidine tag at the C terminus) was incubated with HeLa cells transduced with the ACE2 orthologs. The binding of S1-His to ACE2 was then detected and quantified by flow cytometry analysis (Fig. 2A and Fig. S1). As shown, the binding efficiency of S1 of Omicron (98.63%) was higher than that of D614G (91.63%), suggesting that S1 of Omicron binds human ACE2 with a higher affinity (Fig. 2B). Meanwhile, we also tested whether the binding of the spike protein of Omicron to mouse, marmoset, and koala ACE2 orthologs was altered. As expected, D614G S1-His could not bind mouse, marmoset, or koala ACE2, as previously reported (2, 17, 26). In contrast, S1-His of Omicron could efficiently bind mouse ACE2 (97.93%) but still failed to bind marmoset and koala ACE2s (Fig. 2B).

10.1128/mbio.00416-23.1 FIG S1 Gating strategy for the determination of the efficiency of binding of ACE2 orthologs to SARS-CoV-2 S1-His protein. (A) Schematic for testing the efficiency of ACE2 binding to the recombinant viral RBD-His protein. (B to E) The main cell population was identified and gated on forward scatter (FSC) and side scatter. Single cells were further gated on FSC-A and FSC-H. The gated cells were plotted by fluorescein isothiocyanate A (FITC-A) (zsGreen, as the ACE2-expressing population) and phycoerythrin A (PE-A) (S1-His-bound population). The FITC-A-positive cell population is plotted to show the S1-His-positive population as described in the legend of Fig. 2B. The binding efficiency was defined as the percentage of S1-His-binding cells among the zsGreen-positive cells. Shown are fluorescence-activated cell sorting (FACS) plots representative of those that have been used for the calculations of the efficiencies of binding of ACE2 variants to S1-His. This experiment was independently repeated three times, with similar results. Download FIG S1, TIF file, 0.7 MB. Copyright © 2023 Ren et al. 2023 Ren et al. https://creativecommons.org/licenses/by/4.0/ This content is distributed under the terms of the Creative Commons Attribution 4.0 International license.

To further quantify the direct binding of the RBD of D614G or Omicron to human or mouse ACE2 orthologs, we purified recombinant human and mouse ACE2s as well as D614G and Omicron variant RBDs (Fig. S2) and directly assayed protein binding *in vitro* by surface plasmon resonance (SPR) analysis (Fig. 2C). The dissociation constant (*K_D_*) for human ACE2 binding to the D614G RBD was 8.71 nM, while that of the Omicron RBD was 3.45 nM, about 2.5-fold higher than that of the D614G RBD. As expected, the D614G RBD could not bind mouse ACE2, but strikingly, the Omicron RBD could bind mouse ACE2 with a *K_D_* of 97.64 nM.

10.1128/mbio.00416-23.2 FIG S2 The N-terminal peptidase domain of human and mouse ACE2s (residues Met1 to Asp615) and the WT or Omicron RBD (residues Thr333 to Pro527) were expressed and purified as described in Materials and Methods. The purified proteins were analyzed by SDS-PAGE with Coomassie blue staining. Download FIG S2, TIF file, 0.2 MB. Copyright © 2023 Ren et al. 2023 Ren et al. https://creativecommons.org/licenses/by/4.0/ This content is distributed under the terms of the Creative Commons Attribution 4.0 International license.

Altogether, our results demonstrate that the spike protein of Omicron has evolved to enhance its binding affinity for human ACE2, indicating the increased transmissibility of the Omicron variant. Remarkably, it also gained the function of binding to mouse ACE2, with the potential to extend SARS-CoV-2’s host range.

### Omicron’s spike protein gains the function of engaging mouse ACE2, but not marmoset and koala ACE2s, for cell entry.

**FIG 3:**
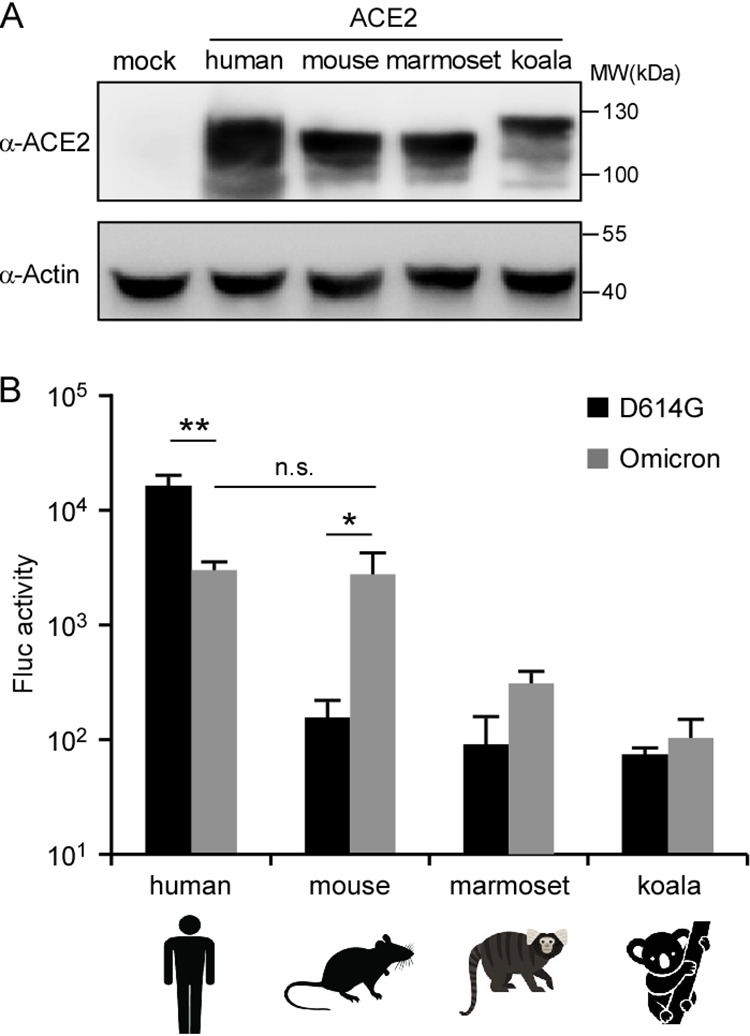
Enhanced cell entry of Omicron spike-pseudotyped virions by utilization of mouse ACE2. (A) Representative immunoblots of HeLa cells transduced with lentiviruses expressing the indicated FLAG-tagged ACE2 orthologs for the detection of ACE2 expression. Actin served as the loading control. These experiments were independently performed twice, with similar results. MW, molecular weight marker. (B) HeLa cells transduced with ACE2 orthologs were infected with the indicated SARS-CoV-2 pseudoparticles. Two days after infection, cells were lysed, and firefly luciferase (Fluc) activity was determined. All infections were performed in triplicate, and the data are representative of results from two independent experiments (means ± SD). n.s., no significance; *, *P* < 0.05; **, *P* < 0.01. Significance was assessed by one-way ANOVA.

To functionally characterize the engagement of ACE2 orthologs by Omicron spike for cell entry, we produced murine leukemia virus (MLV)-pseudotyped virus particles containing a firefly luciferase reporter gene with the spike proteins of the D614G and Omicron variants incorporated on their surface. The yields of the two pseudotypes were semiquantified and normalized by the detection of MLV capsid (anti-p30) antigen using a Western blot assay (Fig. S3). HeLa cells expressing human, mouse, marmoset (New World monkey), or koala ACE2 orthologs (Fig. 3A) were then inoculated with equal amounts of these pseudoparticles; after 48 h, the cells were lysed, and the luciferase activity was assayed as a measure of virus entry (Fig. 3B). The D614G and Omicron spike proteins mediated viral entry into HeLa-human ACE2 cells with varied abilities, and Omicron showed an ~80% reduction compared with D614G. To understand the genetic determinants of Omicron spike responsible for the decreased efficiency of entry into HeLa-human ACE2 cells, we generated the following chimeric recombinant SARS-CoV-2 spike genes carrying the NTD, the RBD, subdomain 2 (SD2), or heptad repeat 1 (HR1) of the D614G spike gene in the backbone of the Omicron spike gene: Omi-NTD(D614G), Omi-RBD(D614G), Omi-SD2(D614G), and Omi-HR1(D614G). We then tested the entry of these pseudoparticles carrying these spike mutants into HeLa-human ACE2 cells (Fig. S4A to C). Our results showed that the RBD and the HR1 region are key determinants of the decreased cell entry of Omicron spike into HeLa-human ACE2 cells.

10.1128/mbio.00416-23.3 FIG S3 Immunoblot analysis of pseudotyped viruses of the D614G and Omicron variants. Full-length spike (S0) and S2 proteins are indicated. HEK293T cells were cotransfected with the retroviral vectors pTG-MLV-Fluc, pTG-MLV-Gag-pol, and pcDNA3.1 expressing the SARS-CoV-2 spike gene (D614G or Omicron). Pseudotyped viral particles were collected for Western blot assays to probe spike protein and MLV capsid p30. The results shown are representative of those obtained in at least two independent experiments. Download FIG S3, TIF file, 0.2 MB. Copyright © 2023 Ren et al. 2023 Ren et al. https://creativecommons.org/licenses/by/4.0/ This content is distributed under the terms of the Creative Commons Attribution 4.0 International license.

10.1128/mbio.00416-23.4 FIG S4 Entry of chimeric recombinant Omicron spike-pseudotyped viruses into HeLa-human ACE2 cells. (A) Schematic overview of SARS-CoV-2 spike proteins of Omicron (B.1.1.529) and the chimeric mutants carrying regions of the D614G spike gene. NTD, N-terminal domain; RBD, receptor-binding domain; SD1, subdomain 1; SD2, subdomain 2; FP, fusion peptide; HR1, heptad repeat 1; HR2, heptad repeat 2; TM, transmembrane region; CT, cytoplasmic tail. (B) HeLa-human ACE2 cells were infected with the indicated SARS-CoV-2 pseudoparticles. Two days after infection, cells were lysed, and luciferase activity was determined. All infections were performed in triplicate, and the data are representative of results from two independent experiments (means ± SD). n.s., no significance; *, *P* < 0.05; **, *P* < 0.01; ***, *P* < 0.001. Significance was assessed by one-way ANOVA. (C) Immunoblot analysis of pseudotyped viruses. Full-length spike (S0) and S2 proteins are indicated. HEK293T cells were cotransfected with the retroviral vectors pTG-MLV-Fluc, pTG-MLV-Gag-pol, and pcDNA3.1 expressing SARS-CoV-2 spike genes, as indicated. Pseudotyped viral particles were collected for Western blot assays to probe spike protein and MLV capsid p30. The results shown are representative of those obtained in at least two independent experiments. Download FIG S4, TIF file, 0.2 MB. Copyright © 2023 Ren et al. 2023 Ren et al. https://creativecommons.org/licenses/by/4.0/ This content is distributed under the terms of the Creative Commons Attribution 4.0 International license.

**FIG 4:**
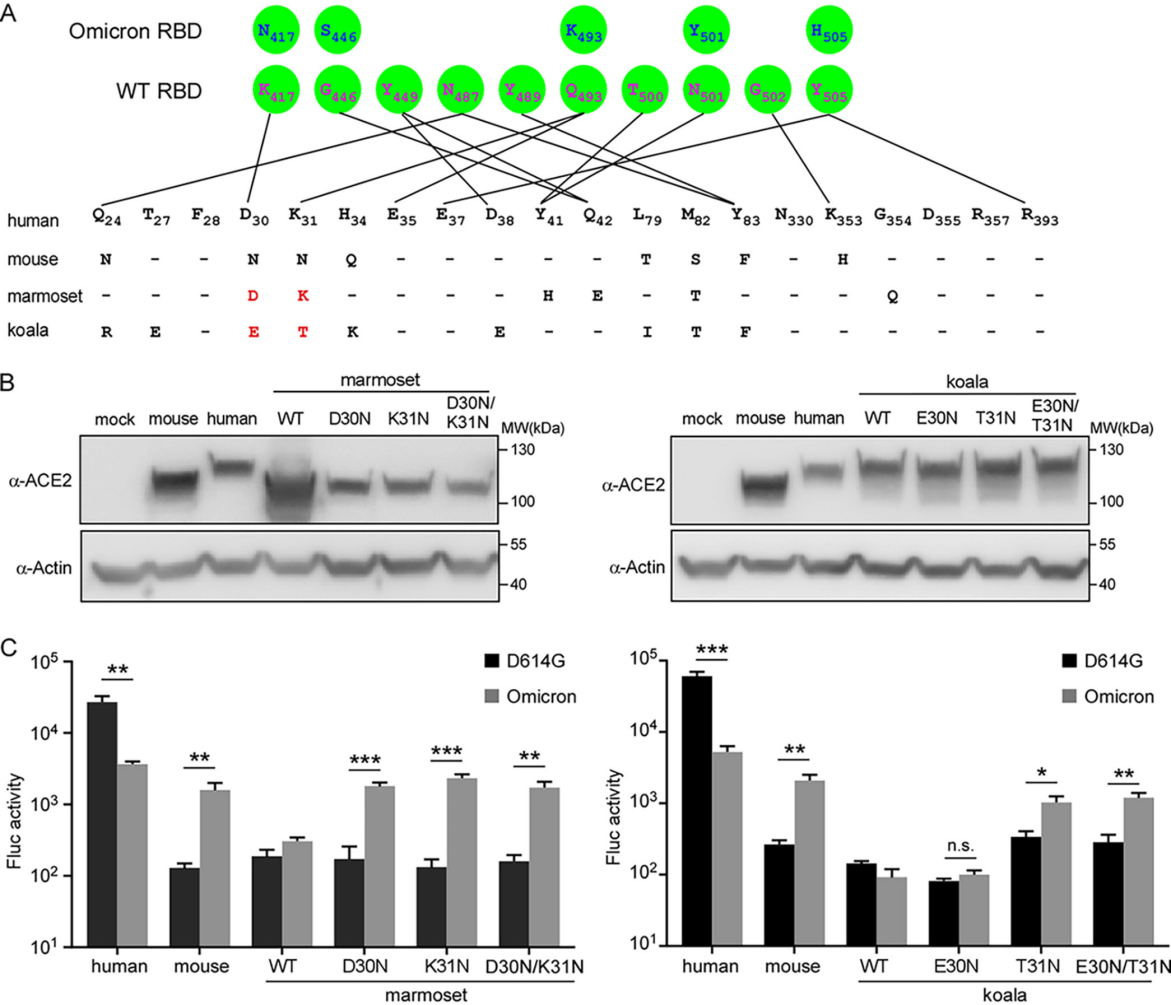
Evaluation of the function of marmoset and koala ACE2 mutants in mediating the entry of SARS-CoV-2 pseudoparticles. (A) Alignment of the residues of human (Homo sapiens) (NCBI reference sequence accession no. NM_001371415.1), mouse (Mus musculus) (NCBI reference sequence accession no. NM_001130513.1), marmoset (Callithrix jacchus) (NCBI reference sequence accession no. XP_008987241.1), and koala (Phascolarctos cinereus) (NCBI reference sequence accession no. XP_020863153.1) ACE2s at the interface of ACE2 and the SARS-CoV-2 spike protein. Residues 30 and 31 in marmoset and koala ACE2s are shown in red. (B) Representative immunoblots of HeLa cells transduced with lentiviruses expressing ACE2 orthologs and mutants, as indicated. Actin was used as the loading control. These experiments were independently performed twice, with similar results. (C) HeLa cells transduced with ACE2 orthologs and mutants were infected with the indicated SARS-CoV-2 pseudoparticles. Two days after infection, cells were lysed, and luciferase activity was determined. All infections were performed in triplicate, and the data are representative of results from two independent experiments (means ± SD). n.s., no significance; *, *P* < 0.05; **, *P* < 0.01; ***, *P* < 0.001. Significance was assessed by one-way ANOVA.

In contrast, the spike protein of Omicron significantly enhanced entry into HeLa-mouse ACE2 cells, which are resistant to D614G spike protein-driven cell entry. However, similar to D614G spike, the Omicron spike protein exhibited a limited ability to utilize marmoset or koala ACE2 for cell entry. To explore the potential reason for this, we aligned the residues of mouse, marmoset, and koala ACE2 orthologs at the binding interface with the viral RBD (27) and compared the residues of ACE2 that could form hydrogen bonds with the residues of the RBD. We hypothesize that the differences at the 30th and 31st residues across mouse, marmoset, and koala ACE2s were responsible for the distinct receptor activities with Omicron spike (Fig. 4A). To test this, we generated a set of HeLa cells expressing marmoset and koala ACE2 mutants bearing single or double residues of mouse ACE2, marmoset ACE2(D30N), marmoset ACE2(K31N), marmoset ACE2(D30N/K31N), koala ACE2(E30N), koala ACE2(T31N), and koala ACE2(D30N/K31N) (Fig. 4B), and tested their function in mediating the cell entry of D614G- or Omicron-pseudotyped viruses (Fig. 4C). Our data showed that Omicron-pseudotyped virus could significantly utilize marmoset ACE2(D30N), marmoset ACE2(K31N), marmoset ACE2(D30N/K31N), koala ACE2(T31N), and koala ACE2(D30N/K31N), but not the others, for cell entry. Thus, these results demonstrated that the 30th and 31st residues were responsible for the inefficacy of marmoset ACE2 to bind to omicron spike; as for koala ACE2, the 31st residue is responsible for this phenotype. Taken together, our results demonstrate that the spike protein of Omicron has been altered in utilizing human ACE2 and animal orthologs for cell entry. The Omicron variant has been compromised in using human ACE2 for cell entry, even with an enhanced binding affinity for human ACE2. Remarkably, the Omicron variant gained the function of utilizing mouse ACE2 orthologs, which cannot be engaged with the D614G virus, for cell entry, with the potential to extend its host range to this species.

### The Omicron variant can establish infection in WT mice.

**FIG 5:**
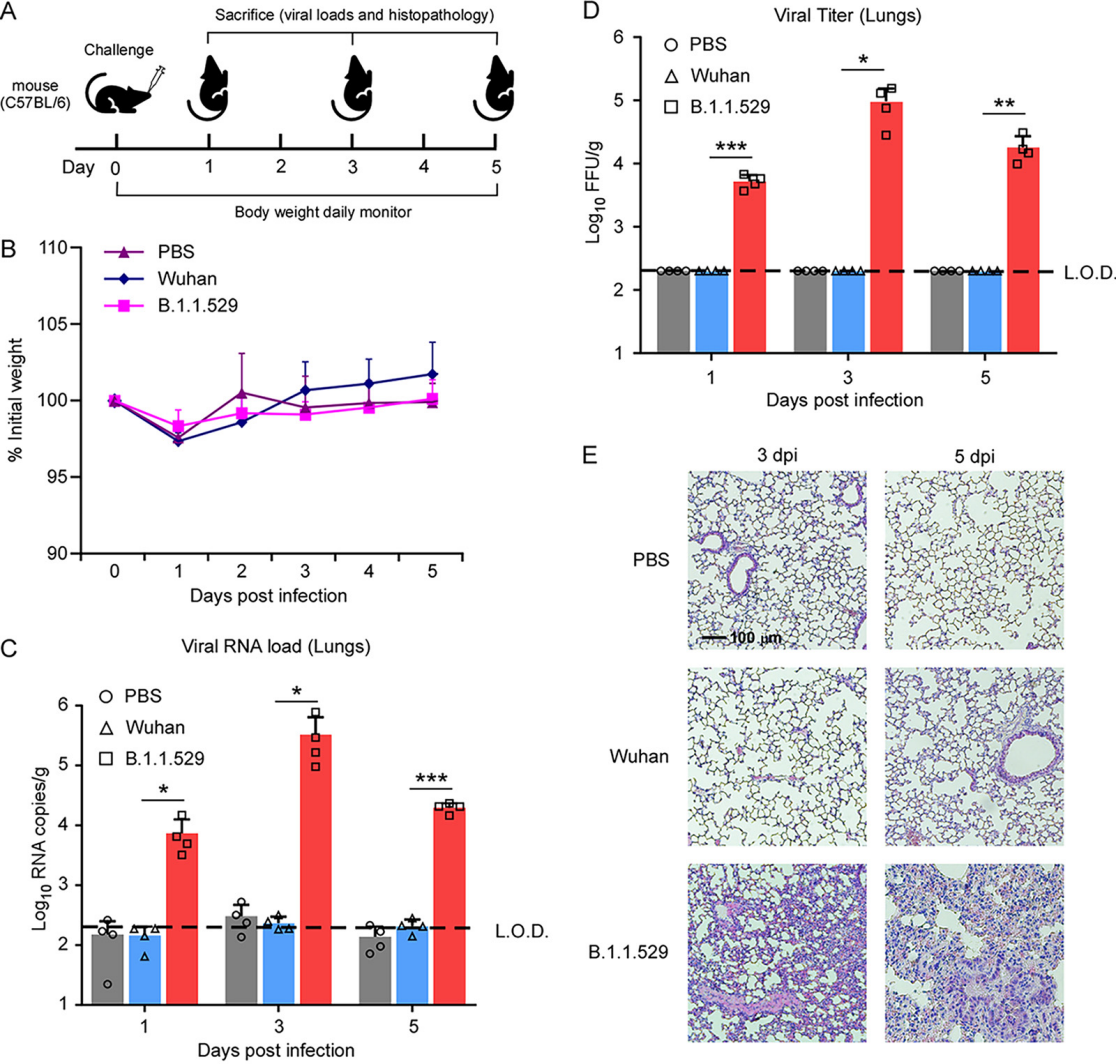
The SARS-CoV-2 Omicron variant can infect C57BL/6 mice. (A) Schematic representation of the experimental timeline. (B) Percent weight changes at different days of postinfection, normalized to the body weight at the time of infection. Bars represent means ± SD. All data are from 2 independent experiments with 10 animals per group. (C and D) Viral RNA loads or infectious viral particles in the lungs of mice infected with 10^5^ TCID_50_ of WT SARS-CoV-2, the Omicron variant, or PBS at different days postinfection, expressed as log_10_ viral RNA copies per gram of lung tissue or log_10_ FFU per gram of lung tissue. Individual data and median values are presented. The dashed line represents the limit of detection (L.O.D.). Data were analyzed by the Mann-Whitney U test. *, *P* < 0.05; **, *P* < 0.01; n.s. not significant. (E) Representative H&E-stained images of lung sections of mice infected with the WT or the Omicron variant at 3 days postinfection (dpi) (left) or 5 days postinfection (right).

As Omicron-pseudotyped viral particles could utilize mouse ACE2 for cell entry, we decided to assess the infection of mice *in vivo* using an authentic Omicron variant. For this purpose, we intranasally infected wild-type C57BL/6 mice with a SARS-CoV-2 ancestor virus (WT) (WuHan IVCAS 6.7512 strain), the Omicron variant (Omicron IVCAS 6.7600 strain) (1 × 10^5^ 50% tissue culture infective doses [TCID_50_]/mouse), or phosphate-buffered saline (PBS) (negative control). Mice were weighed daily and sacrificed on day 1, 3, or 5 postinfection to harvest nasal turbinates and lung tissues (Fig. 5A). The viral RNAs in the nasal turbinates and lungs of mice were quantified by a reverse transcription-quantitative PCR (RT-qPCR) assay, and infectious viruses in the lungs were titrated by a focus-forming unit (FFU) assay. Actually, no obvious body weight loss or clinical symptoms were observed in infected mice from all three groups (Fig. 5B). As expected, WT virus RNA and infectious viruses were hardly detected, and no apparent histopathological changes were observed in the lungs of C57BL/6 mice (Fig. 5C to E). In contrast, Omicron variant RNA and viruses in the lungs of C57BL/6 mice could be readily detected at 1, 3, and 5 days postinfection, with viral RNA abundances ranging from 10^4^ to 10^6^ copies per g of tissue and levels of infectious virus ranging from 10^3^ to 10^5^ FFU per g of tissue. Of note, Omicron RNA was not detected in the turbinates (Fig. S5). Additionally, pulmonary edema with fluid exudation and monocyte infiltration were obviously observed in the lung sections both 3 and 5 days after infection by the Omicron variant but not in those of PBS- and WT virus-inoculated mice (Fig. 5E). Thus, these data indicate that mutations in the Omicron strain confer the ability to replicate efficiently in the lungs of wild-type C57BL/6 mice.

10.1128/mbio.00416-23.5 FIG S5 SARS-CoV-2 WT or Omicron variant infection of C57BL/6 mice. Viral RNA loads in the turbinates of mice infected with 10^5^ TCID_50_ of WT SARS-CoV-2, the Omicron variant, or PBS at different days postinfection (pi) are expressed as log_10_ viral RNA copies per gram of tissue. The dashed line represents the limit of detection (L.O.D.). Data were analyzed with the Mann-Whitney U test. n.s., no significance. Download FIG S5, TIF file, 0.03 MB. Copyright © 2023 Ren et al. 2023 Ren et al. https://creativecommons.org/licenses/by/4.0/ This content is distributed under the terms of the Creative Commons Attribution 4.0 International license.

## DISCUSSION

In this study, we have shown that Omicron spike exhibited significant evasion of neutralizing antibodies elicited by three doses of an inactivated vaccine (Fig. 1B) but remained sensitive to inhibition by an ACE2 decoy receptor (Fig. 1C). Meanwhile, the Omicron spike protein has increased its binding affinity for human ACE2; remarkably, it has gained the function to interact with mouse ACE2 for cell entry both *in vitro* and *in vivo* (Fig. 2, 3, and 5), suggesting that Omicron has expanded its host range to mice. It is conceivable that the increases in antibody evasion and human ACE2 binding as well as the expanded host range are potentially factors that contribute to the increase in the transmissibility of the Omicron variant.

Compared with D614G virus, Omicron has exhibited resistance to vaccine-induced neutralization, and also, the Omicron variant evolved to increase its ability to bind human ACE2 despite these extensive mutations (Fig. 1 and 2), indicating that the Omicron variant has evolved to balance an increase in escape from neutralization with its ability to interact with ACE2. Other VOCs such as Alpha, Beta, Gamma, and Delta have similar evolutionary patterns in that they increased their abilities to bind human ACE2 and escape neutralizing-antibody-based immunity (14, 28–32). However, they remain sensitive to ACE2 decoy receptor. Thus, the ACE2 decoy receptor antiviral strategy represents a broad-spectrum antiviral countermeasure against current and future VOCs as well as other coronaviruses that utilize ACE2 as a receptor. In addition, it has been demonstrated that human ACE2 peptidase activity and viral receptor activity could be uncoupled (33, 34); thus, it is possible that enzymatically inactivated ACE2 can be developed as an antiviral in the clinic to avoid potential ACE2 side effects mediated by its enzymatic activity.

Actually, a phase II clinical trial with a recombinant human ACE2 decoy receptor (APN01) for the treatment of COVID-19 patients by intravenous administration (ClinicalTrials.gov identifier NCT04335136) failed to meet the primary outcomes (all-cause death, invasive mechanical ventilation for up to 28 days, or hospital discharge). Therefore, an aerosol formulation of the ACE2 decoy receptor via an inhaler is currently being tested in a phase I study (ClinicalTrials.gov identifier NCT05065645) (35). In addition, soluble ACE2 decoy proteins have been bioengineered. To prolong the duration of action and increase the binding affinity for SARS-CoV-2, the ACE2 decoy protein fused with an albumin-binding domain (ABD), which is linked via a dimerization motif dodecapeptide (DDC) (ACE2 618-DDC-ABD), was developed, and the ACE2 618-DDC-ABD protein exhibited high efficacy for the protection of K18-hACE2 mice from lethal SARS-CoV-2 infection (36). Recently, vectored immunoprophylaxis for SARS-CoV-2 using adeno-associated virus (AAV) or lentiviral vectors expressing an ACE2 decoy receptor was reported, and the administration of ACE2 decoy-expressing AAV could protect mice from high-titer SARS-CoV-2 and Omicron variant challenges (37). Decoy-vectored immunoprophylaxis could be valuable for immunocompromised individuals for whom vaccination is not practical, and this approach is expected to remain active against newly emerging variants. It is conceivable that an ACE2 decoy receptor that is engineered to confer binding affinity and *in vivo* stability could be highly useful for therapeutic or prophylactic purposes.

Several strains such as B.1.1.7 (Alpha), B.1.351 (Beta), and P.1 (Gamma) acquired a mouse-adapting substitution (N501Y) in the RBD of the spike protein (9, 38, 39), which enables the utilization of mouse ACE2 for cell entry *in vitro* and *in vivo* (9, 40). Omicron spike also harbors the N501Y mutation, suggesting that Omicron could infect WT mice without human ACE2 expression. We found that Omicron has evolved to bind mouse ACE2 (*K_D_* = 97.64 nM) for cell entry (Fig. 2A to C and Fig. 3) and establish infection in C57BL/6 mice (Fig. 5). Interestingly, Omicron RNA could be detected in the lungs, but not the nasal turbinates, of C57BL/6 mice (Fig. 5D; see also Fig. S5 in the supplemental material), which is different from previous observations that Omicron could be detected in both the lungs and turbinates (19, 20). Compared with previous studies (19, 20), the mouse strains, ages, sexes, and virus doses varied dramatically, which may lead to the different observations. In line with our observations, a recent study compared infections by ancestral SARS-CoV-2 and the Omicron variant in K18-hACE2 mice, and they found that Omicron viral antigen was hardly detected in the nasal turbinates (41), which could partially explain the attenuated pathogenicity of the Omicron variant. Taken together, these results suggest that the Omicron variant has extended its host range to mice, raising the potential risk of mice becoming reservoirs for SARS-CoV-2, and the virus could potentially spill back to humans as mice live in proximity to humans.

The Omicron variant has already become the dominant variant in many countries, although it causes attenuated diseases compared with previous variants (19, 42). A third dose (booster) of an mRNA vaccine (22, 43), subunit vaccine, or inactivated vaccine (44, 45) may increase the level of cross-neutralizing antibodies to the Omicron variant based on a growing body of evidence and the present study; however, the rapid development of new, variant-specific vaccines is warranted. In addition, as Omicron has evolved to utilize mouse ACE2 for cell entry *in vitro* and *in vivo*, we recommend that the host range should be closely monitored along with the continued evolution of SARS-CoV-2 variants to prevent future zoonosis-associated outbreaks.

## MATERIALS AND METHODS

### Ethics statement.

This animal experiment protocol (SARS-CoV-2 infection of C57BL/6 mice) has been approved by the Animal Ethics Committee of the Wuhan Institute of Virology, Chinese Academy of Sciences.

### Cell culture and SARS-CoV-2.

HEK293T (ATCC CRL-3216; American Type Culture Collection [ATCC], Manassas, VA), Vero E6 (Cell Bank of the Chinese Academy of Sciences, Shanghai, China), and HeLa (a kind gift from Nian Liu at Tsinghua University) cells were maintained in Dulbecco’s modified Eagle medium (DMEM) (Gibco, NY, USA) supplemented with 10% (vol/vol) fetal bovine serum (FBS), 10 mM HEPES, 1 mM sodium pyruvate, 1× nonessential amino acids, and 50 IU/mL penicillin-streptomycin in a humidified 5% (vol/vol) CO_2_ incubator at 37°C. Cells were tested routinely and found to be free of mycoplasma contamination. The SARS-CoV-2 ancestor virus (WuHan IVCAS 6.7512 strain) isolated from a COVID-19 patient and the Omicron variant (Omicron IVCAS 6.7600 strain) were provided by the National Virus Resource Center. All of the viruses were propagated and titers were determined in Vero E6 cells. All experiments involving virus infections were performed in the biosafety level 3 facility of the Wuhan Institute of Virology, Chinese Academy of Sciences, following all regulations.

### Plasmids.

cDNAs encoding the human, koala, New World monkey, or mouse ACE2 were synthesized by GenScript and cloned into the pLVX-IRES-zsGreen1 vector (catalog no. 632187; Clontech Laboratories, Inc.) with a C-terminal FLAG tag. All constructs were verified by Sanger sequencing.

### Lentivirus production.

Vesicular stomatitis virus G protein (VSV-G)-pseudotyped lentiviruses expressing ACE2 orthologs tagged with FLAG at the C terminus were produced by the transient cotransfection of the third-generation packaging plasmids pMD2G (Addgene plasmid 12259) and psPAX2 (Addgene plasmid 12260) and the transfer vector with VigoFect DNA transfection reagent (Vigorous Biotechnology) into HEK293T cells. The medium was changed at 12 h posttransfection. The supernatants were collected 24 and 48 h after transfection, pooled, passed through a 0.45-μm filter, and frozen at −80°C.

### Analysis of surface ACE2 binding by an RBD-His assay.

HeLa cells were transduced with lentiviruses expressing the ACE2 variants for 48 h. The cells were collected with TrypLE (catalog no. 12605010; Thermo) and washed twice with cold PBS. Live cells were incubated with the recombinant proteins S1-His (catalog no. 40591-V08H for D614G and 40592-V08H121 for Omicron; Sino Biological) (1 μg/mL) at 4°C for 30 min. After washing, cells were stained with anti-His-phycoerythrin (PE) (clone GG11-8F3.5.1) (catalog no. 130-120-787; Miltenyi Biotec) for 30 min at 4°C. Cells were then washed twice and subjected to flow cytometry analysis (Attune NxT; Thermo). Binding efficiencies are expressed as the percentage of cells positive for RBD-His among the zsGreen-positive cells (ACE2-expressing cells).

### Surface plasmon resonance analysis.

The D614Gor Omicron SARS-CoV-2 RBD (residues Arg319 to Phe541) and the N-terminal peptidase domain of human or mouse ACE2 (residues Ser19 to Asp615) were expressed using the Bac-to-Bac baculovirus system (Invitrogen) as described previously (46). ACE2 was immobilized on a CM5 chip (GE Healthcare) to a level of around 500 response units using a Biacore T200 instrument (GE Healthcare) and running buffer (10 mM HEPES [pH 7.2], 150 mM NaCl, and 0.05% Tween 20). Serial dilutions of the SARS-CoV-2 RBD were flowed through with a range of concentrations. The resulting data were fit to a 1:1 binding model using Biacore Evaluation software (GE Healthcare).

### Production of SARS-CoV-2-pseudotyped virus, determination of viral entry efficiency, and analysis of spike protein cleavage.

To produce virions pseudotyped with the SARS-CoV-2 spike protein, HEK293T cells were transfected with the retroviral vectors pTG-MLV-Fluc, pTG-MLV-Gag-pol, and pcDNA3.1 expressing the SARS-CoV-2 spike gene using VigoFect (Vigorous Biotechnology). Forty-eight hours after transfection, the cell culture supernatant was collected by centrifugation at 3,500 rpm for 10 min, and the supernatant was subsequently aliquoted and stored at −80°C until use. Virus entry was assessed by the transduction of pseudoviruses in cells expressing ACE2 in 48-well plates. After 48 h, intracellular luciferase activity was determined using a luciferase assay system (catalog no. E1500; Promega) according to the manufacturer’s instructions. Luminescence was recorded on a GloMax Discover system (Promega). For the analysis of spike protein cleavage, concentrated pseudoviruses were produced by ultracentrifugation at 100,000 × *g* for 2 h over a 20% sucrose cushion. Western blot detection of SARS-CoV-2 spike protein was performed using a polyclonal spike antibody (catalog no. 40589-T62; Sino Biological).

### Vaccinated human serum and neutralization of pseudotyped virion particles.

Serum samples from individuals who received three doses of a COVID-19 vaccine (Sinovac-CoronaVac or BBIBP-CorV) were collected either 1 to 2 months after the third vaccination (see Table S1 in the supplemental material). Each plasma sample was heat inactivated (56°C for 30 min) and then assayed for neutralization against D614G or Omicron pseudoviruses. Specifically, spike protein-bearing pseudotyped virion particles were preincubated for 1 h at 37°C with diluted plasma samples obtained from vaccinated individuals, before the mixtures were inoculated onto HeLa-ACE2 cells. After 48 h of inoculation, the transduction efficiency was determined by a luciferase activity assay. A nonlinear curve and 50% pseudotyped-virus-neutralizing antibody titer (pVNT_50_) values were generated using GraphPad Prism. This study was approved by the Institutional Review Board of Tsinghua University (approval no. 20210040).

### Recombinant ACE2-Ig protein expression and purification.

ACE2-Ig, a recombinant Fc fusion protein of soluble human ACE2 (residues Gln18 to Ser740), was expressed in 293F cells and purified using protein A affinity chromatography as described in our previous study (24).

### SARS-CoV-2 infection of C57BL/6 mice.

Seven-week-old male C57BL/6 mice were infected intranasally with WT SARS-CoV-2 or the Omicron variant (1 × 10^5^ TCID_50_ per mouse; 50-μL inoculum per mouse). The mice were weighed daily for 7 days and were then euthanized and perfused extensively with PBS. Nasal turbinates and lung tissues were harvested and fixed in 4% paraformaldehyde (PFA) for 48 h. Tissues were embedded in paraffin for sectioning and stained with hematoxylin and eosin (H&E) to assess tissue morphology. RNAs from nasal turbinates or lung tissues were extracted using the TRIzol reagent (catalog no. 15596018; Thermo Fisher). Viral or host RNA levels were determined using the TaqPath one-step RT-qPCR master mix (catalog no. A15299; Thermo Fisher) on a CFX Connect real-time system (Bio-Rad). A standard curve was produced using serial 10-fold dilutions of *in vitro*-transcribed RNA of the S gene. The viral burden was expressed on a log_10_ scale as viral RNA copies per gram of tissue. Primers used are as follows: WT-RBD-qF1 (5′-CAA TGG TTT AAC AGG CAC AGG-3′), WT-RBD-qR1 (5′-CTC AAG TGT CTG TGG ATC ACG-3′), Omic-RBD-qF1 (5′-CAA TGG TTT AAA AGG CAC AGG-3′), and Omic-RBD-qF1 (5′-CTC AAG TGT CTG TGG ATC ACG-3′).

### Statistical analysis.

Analysis of the statistical significance of the data was performed using GraphPad Prism version 9 (GraphPad Software). One-way analysis of variance (ANOVA) with Tukey’s honestly significant difference (HSD) test was used to test the statistical significance of differences between the different group parameters. *P* values of less than 0.05 were considered statistically significant.
