# Supplementary figures and images for: Evolution of Immune Evasion and Host Range Expansion by the SARS-CoV-2 B.1.1.529 (Omicron) Variant
Source: mBio. 2023 Apr 3;14(2):e00416-23. doi: 10.1128/mbio.00416-23 (PMC10127688; doi:10.1128/mbio.00416-23)

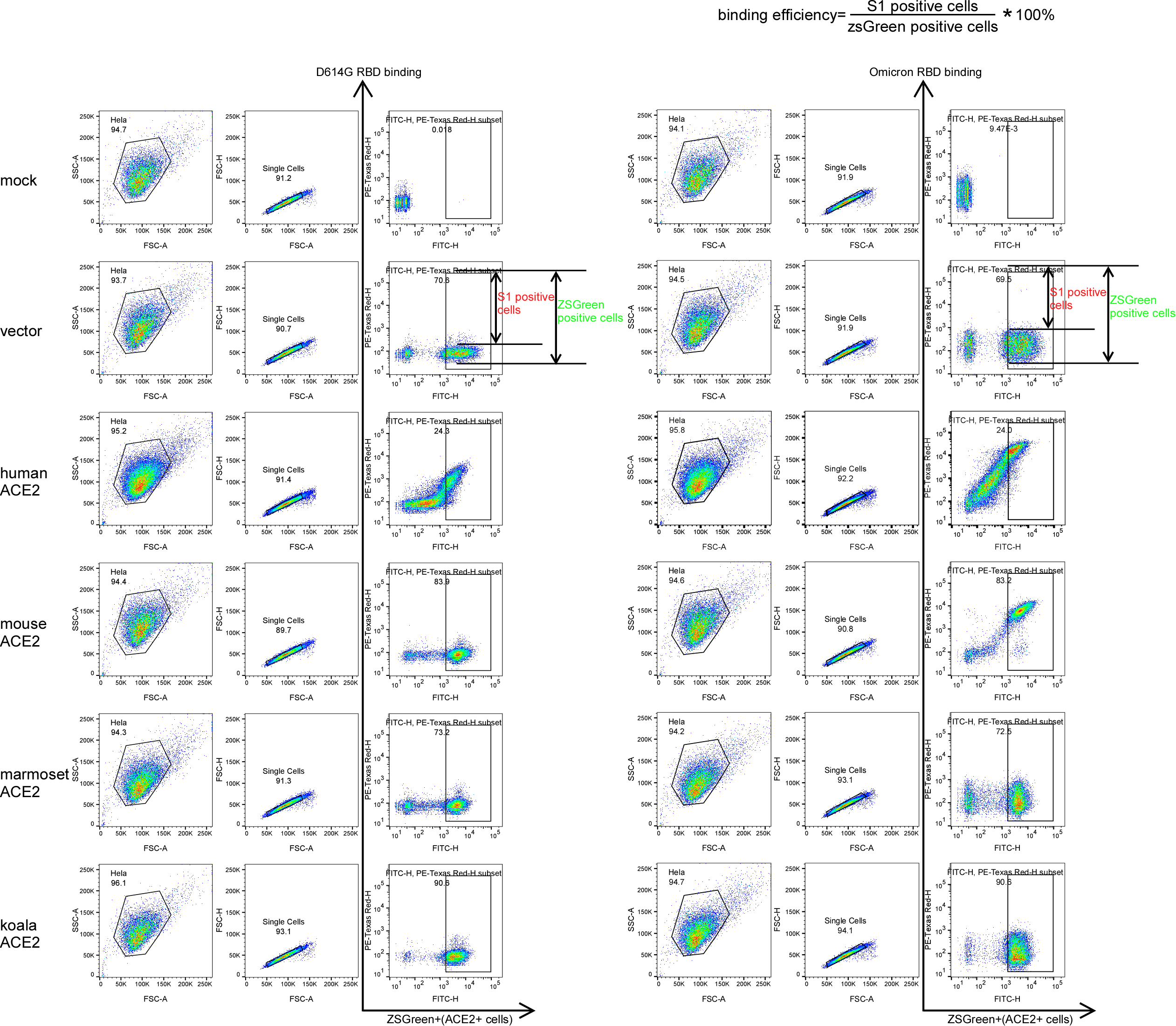

Supplement: FIG S1 [file mbio.00416-23-s0001.tif]

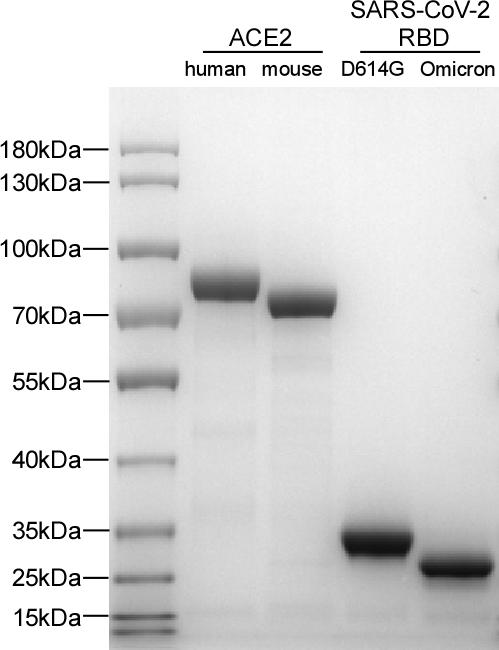

Supplement: FIG S2 [file mbio.00416-23-s0002.tif]

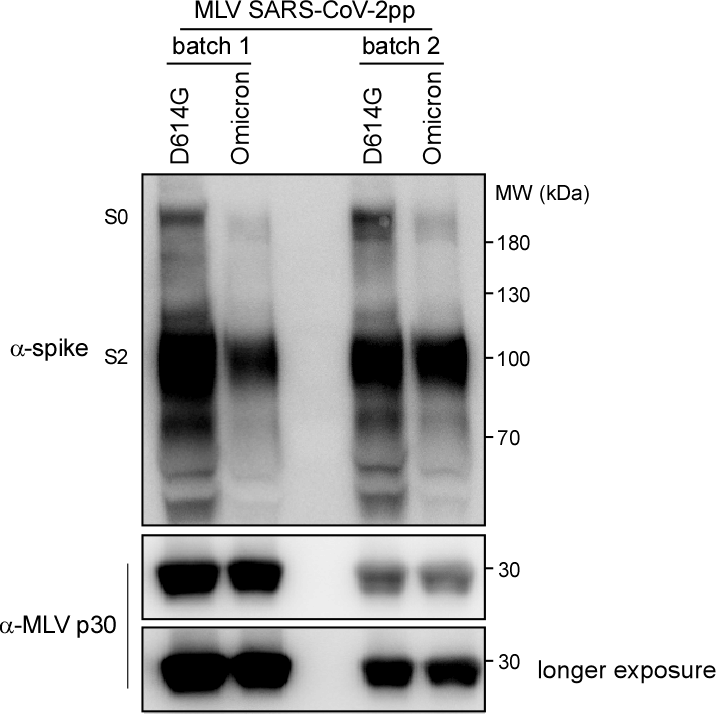

Supplement: FIG S3 [file mbio.00416-23-s0003.tif]

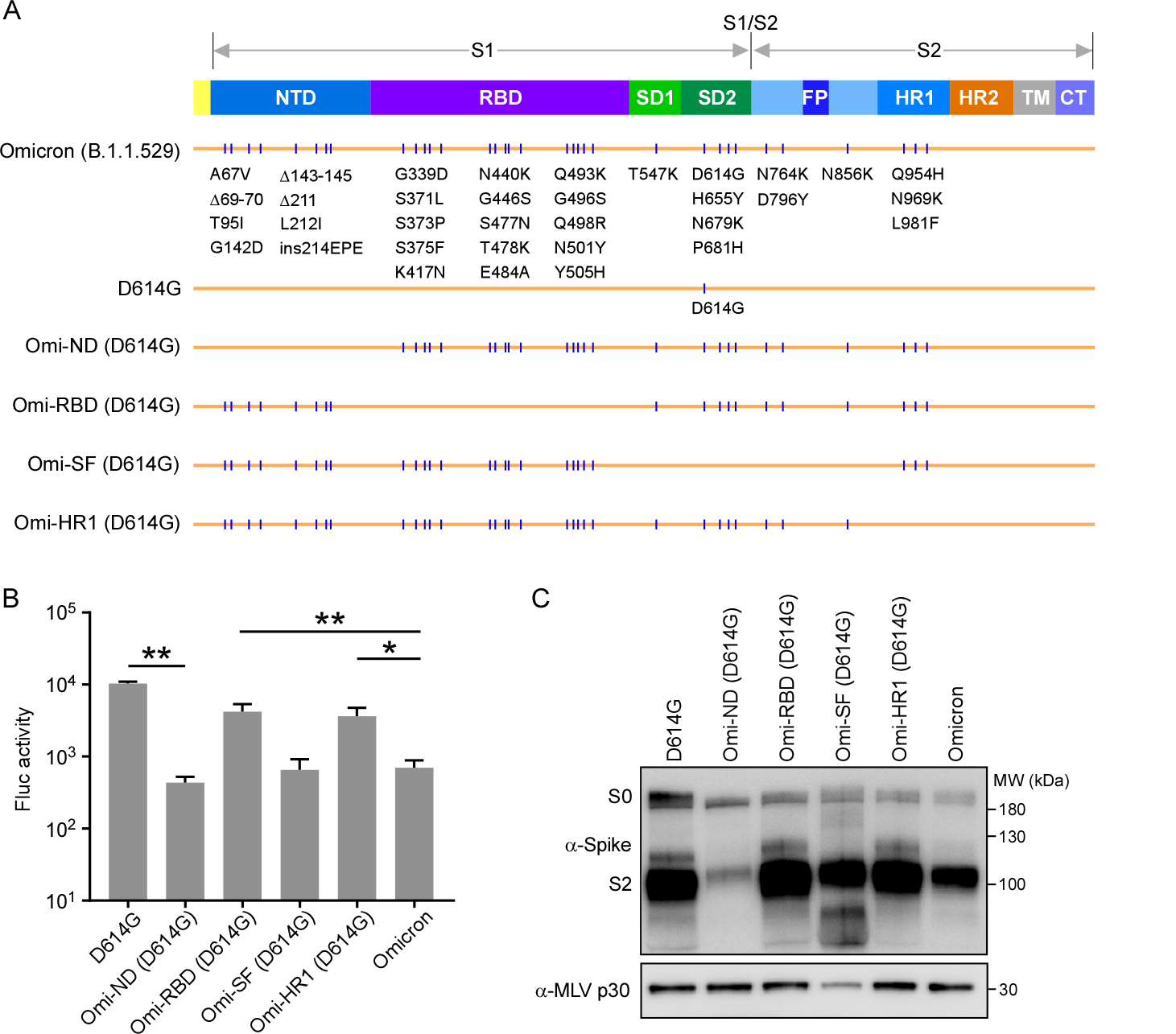

Supplement: FIG S4 [file mbio.00416-23-s0004.tif]

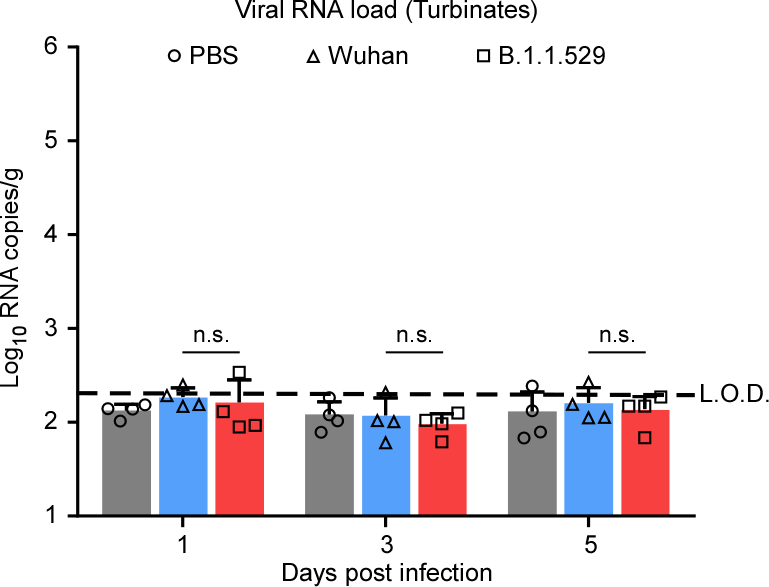

Supplement: FIG S5 [file mbio.00416-23-s0005.tif]
